# Pilot-Scale Testing of UV-A Light Treatment for Mitigation of NH_3_, H_2_S, GHGs, VOCs, Odor, and O_3_ Inside the Poultry Barn

**DOI:** 10.3389/fchem.2020.00613

**Published:** 2020-07-31

**Authors:** Myeongseong Lee, Peiyang Li, Jacek A. Koziel, Heekwon Ahn, Jisoo Wi, Baitong Chen, Zhanibek Meiirkhanuly, Chumki Banik, William S. Jenks

**Affiliations:** ^1^Department of Animal Biosystems Sciences, Chungnam National University, Daejeon, South Korea; ^2^Department of Agricultural and Biosystems Engineering, Iowa State University, Ames, IA, United States; ^3^Department of Chemistry, Iowa State University, Ames, IA, United States

**Keywords:** air pollution, odor, indoor air quality, ammonia, emissions, poultry production, photocatalysis, concentrated animal feeding operations

## Abstract

Poultry farmers are producing eggs, meat, and feathers with increased efficiency and lower carbon footprint. Technologies to address concerns about the indoor air quality inside barns and the gaseous emissions from farms to the atmosphere continue to be among industry priorities. We have been developing and scaling up a UV air treatment that has the potential to reduce odor and other gases on the farm scale. In our recent laboratory-scale study, the use of UV-A (a less toxic ultraviolet light, a.k.a. “black light”) and a special TiO_2_-based photocatalyst reduced concentrations of several important air pollutants (NH_3_, CO_2_, N_2_O, O_3_) without impact on H_2_S and CH_4_. Therefore, the objectives of this research were to (1) scale up the UV treatment to pilot scale, (2) evaluate the mitigation of odor and odorous volatile organic compounds (VOCs), and (3) complete preliminary economic analyses. A pilot-scale experiment was conducted under commercial poultry barn conditions to evaluate photocatalyst coatings on surfaces subjected to UV light under field conditions. In this study, the reactor was constructed to support interchangeable wall panels and installed on a poultry farm. The effects of a photocatalyst's presence (photocatalysis and photolysis), UV intensity (LED and fluorescent), and treatment time were studied in the pilot-scale experiments inside a poultry barn. The results of the pilot-scale experiments were consistent with the laboratory-scale one: the percent reduction under photocatalysis was generally higher than photolysis. In addition, the percent reduction of target gases at a high light intensity and long treatment time was higher. The percent reduction of NH_3_ was 5–9%. There was no impact on H_2_S, CH_4_, and CO_2_ under any experimental conditions. N_2_O and O_3_ concentrations were reduced at 6–12% and 87–100% by both photolysis and photocatalysis. In addition, concentrations of several VOCs responsible for livestock odor were reduced from 26 to 62% and increased with treatment time and light intensity. The odor was reduced by 18%. Photolysis treatment reduced concentrations of N_2_O, VOCs, and O_3_, only. The initial economic analysis has shown that LEDs are more efficient than fluorescent lights. Further scale-up and research at farm scale are warranted.

## Introduction

Poultry farmers are producing eggs, meat, and feathers with increasing efficiency and a lower carbon footprint. Technologies to address concerns about the air quality inside barns and the gaseous emissions from farms to the atmosphere continue to be among industry priorities. Only ~25% of research on technologies to mitigate emissions from animal production systems has been tested on farms, and scaling up technologies from the laboratory to farm scales has proven to be a challenge. However, farmers prefer technologies that are simple to adopt and low in cost. Therefore, pilot- and farm-scale experiments should be preceded for the technical application on the livestock farm. According to previous studies (Maurer et al., 2016), the treatment of odorous emissions with UV light has the potential to be a relatively simple adaptation to existing and new facilities. Yet many practical questions remain to be addressed to scale up this technology from the laboratory to the farm.

Proposed UV-based methods consist of direct irradiation and photocatalytic treatment. In the former, light is directly absorbed by the target gases (or potentially other photoreactive gases). By contrast, photocatalysis consists of the light being principally absorbed by a photoactive coating with secondary reactions between target gases and reactive intermediates taking place mainly at or near the coated surfaces. UV irradiation treatment has a variety of mitigation effects on the target gas, depending on several parameters. Direct irradiation generally requires shorter wavelength light (e.g., 254 nm), in that few target molecules absorb in the near UV (e.g., 360 nm), whereas photocatalytic treatment can proceed with the longer wavelengths because of choice of coating. Light flux is another obvious parameter. Other critical variables in the real application are the presence of other agents in the gas mixture, such as the degree of humidity, and presence or absence of ozone.

The wavelength of irradiation is an important consideration for the application of UV technology because of its potential effects on humans and animals. The shortest, easily accessible wavelengths (e.g., 185 nm) will cause a build-up of ozone and N_2_O because the light is absorbed by atmospheric O_2_. Traditional bactericidal light sources (often 254 nm) do not cause that problem but would cause rapid sunburn for exposed skin. Near-UV light (often called UV-A) that is sufficient for photocatalysis is also not appropriate for long-term skin exposure but is otherwise the most benign. The most common photocatalyst is nano-particulate TiO_2_, which is chosen for its relatively broad application, comparatively high efficiency, durability, lack of toxicity, and low cost (Hashimoto et al., 2005; Zaleska, 2008; Rockafellow et al., 2012; Schneider et al., 2014).

Photocatalysis is initiated when photons of sufficient energy (i.e., greater than the semiconductor band gap) are absorbed by the TiO_2_ particles, resulting in electron/hole (e^−^/h^+^) generation (Vautier et al., 2001; Schneider et al., 2014; Lee et al., 2018; Maurer and Koziel, 2019). For commonly available TiO_2_, the threshold is roughly 380 nm. Under atmospheric moisture conditions, HO• (hydroxyl) radicals are produced by the interaction of hole (h+) with H_2_O molecular (Vautier et al., 2001; Nakata and Fujishima, 2012; Lee et al., 2018; Maurer and Koziel, 2019). The most common electron sink is molecular oxygen. Either through these reactive intermediates or by direct interaction with the e^−^ or h^+^, the target materials are degraded. Full mineralization (conversion to CO_2−_, H_2_O, and inorganic ions) can usually be achieved through exhaustive treatment. Although the detailed mechanism of photocatalysis varies with different target pollutants and treatment conditions, it is commonly agreed that the primary reactions responsible are these interfacial oxidation and reduction reactions (Abe, 2010; Maeda and Domen, 2010; Nakata and Fujishima, 2012; Schneider et al., 2014).

The applicability of UV photocatalytic technology to the farm has been investigated in previous studies. Photocatalysis based on TiO_2_ has been evaluated to reduce odorous gases and fine particulate concentrations as well as for increased feed conversion rates (Guarino et al., 2008; Costa et al., 2012; Zhu et al., 2017; Maurer and Koziel, 2019; Yang et al., 2020). In addition, optimal conditions and parameters affecting target gas mitigation have been investigated (Lee et al., 2020). Moreover, economic analysis has shown that it is reasonable compared with other technologies (Koziel et al., 2008; Liu et al., 2015).

Here, we report a study of the mitigation of odorous target gases, volatile organic compounds (VOCs), greenhouse gases (GHGs), and odor using UV-A treatment in actual poultry farms. This study was brought up to a pilot scale based on the lessons learned about UV-A performance of the photocatalysis demonstrated in a recent laboratory-scale study (Lee et al., 2020). The results provided evidence that photocatalysis with TiO_2_ coating and UV-A light can reduce gas concentrations of NH_3_ (3–19%), CO_2_ (4%), N_2_O (7–12%), and O_3_ (12–48%) without significant effect on H_2_S and CH_4_. In addition, the mitigation of target gases was generally improved with parameters that sensibly dictate higher effective dosages: the presence of photocatalyst, relative humidity (RH, 12%), higher light intensity, longer treatment time, and low dust accumulation on the photocatalyst surface. However, it was found that the optimum mitigation conditions (RH and dust accumulation) and the effect of parameters (light intensity and treatment time) depend on the type of targeted gas.

Poultry barns manage lighting very precisely owing to bird physiological and production needs. Thus, the poultry industry is generally prepared to consider the adoption of light-based technologies. However, carefully scaled-up studies are still needed to move forward UV-based treatment for air quality improvements without jeopardizing current production practices. To date, no studies report on applying actual photocatalysis technology in real poultry farms. Therefore, the objectives of this research are (1) to scale up the UV treatment to pilot scale in the poultry farm, (2) to evaluate the mitigation of NH_3_, H_2_S, GHGs, odor, and odorous VOCs, and (3) to complete a preliminary economic analysis. In addition, the effects of photocatalyst presence (in comparison with direct photolysis), UV intensity (based on LED vs. fluorescent light sources), and treatment time were studied in the pilot-scale experiments inside a poultry barn.

## Materials and Methods

### Experimental Reactor Setup

**Figure 1:**
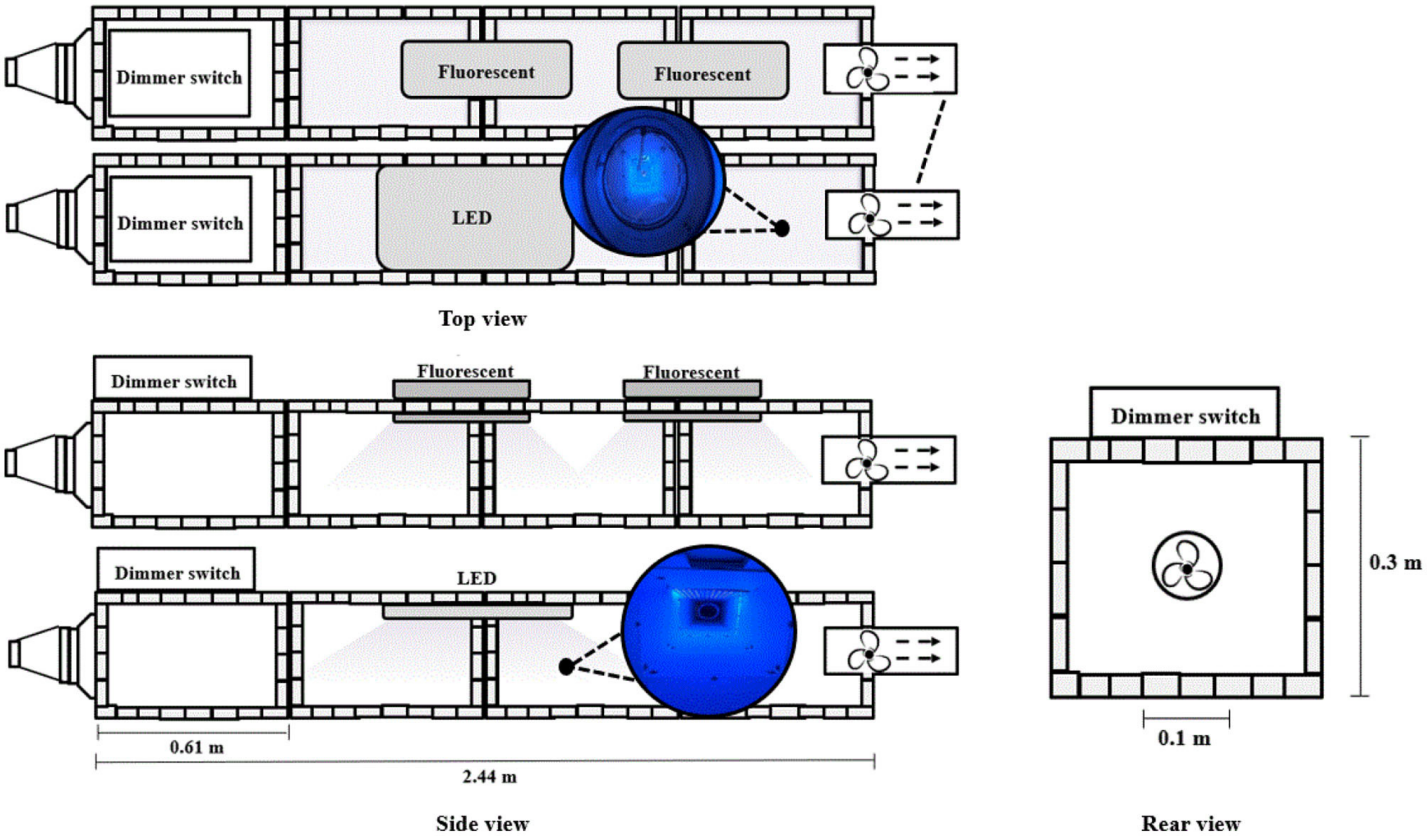
Schematic of pilot-scale UV reactor for mitigation of selected gases inside a poultry barn.

The reactor (2.44 × 0.3 × 0.3 m) was designed with reference to previous research (Maurer and Koziel, 2019) as shown in Figure 1. The frame consisted of a plated steel slotted angle (Lowes, Mooresville, NC, USA) with embossed white fiberglass-reinforced plastic wall panel (Lowes). The frame was constructed to support interchangeable wall panels (0.3 × 0.61 × 0.002 m, regular panel vs. TiO_2_-coated panel). These materials are commonly used as an interior wall in a livestock barn (Maurer and Koziel, 2019). The wall panel's bottom was coated with a photocatalyst (nanostructured anatase TiO_2_ at 10 μg·cm^−2^ from PureTi, Cincinnati, OH, USA). All of the wall panels are changed when testing the effect of photocatalysis from uncoated panels to coated ones. On the front and back panels, a 0.1-m-diameter hole was made to allow airflow in the reactor. The reactor air inlet consisted of two duct reducers with a diameter of 0.20–0.15 m and a diameter of 0.15–0.10 m (Lowes, Mooresville, NC, USA).

A fan was installed in a 0.1 m diameter steel axial duct (Lowes, Mooresville, NC, USA) at the end of the reactor. The fan drew air from the inlet of the reactor to the outlet. The fans were able to adjust the flow rate using the dimmer switch. Therefore, the fan velocity calibration was conducted to adjust the flow rate in the reactor.

The reactor was illuminated with two fluorescents (Spectroline, Westbury, NY, USA) and one LED (ONCE, Plymouth, MN, USA). Both sources emit predominantly at 365 nm. Two fluorescent UV lamps were installed in the middle and 0.15 m behind the center lamp. For the LED-based treatment, one UV lamp was installed in the middle of the reaction.

Measurement of ammonia (NH_3_) and hydrogen sulfide (H_2_S) concentrations, temperature, relative humidity (RH), and ozone (O_3_) were conducted in real time. Temperature and RH were monitored via an 850071 Environmental Quality meter (Sper Scientific, Scottsdale, AZ, USA). Gas samples for GHGs, odorous VOCs, and odor were collected and subsequently analyzed in the laboratory. Samples were collected at the reducer at the front of the reactor and 0.15 m away from the rear board. The measurement of target gases was triplicated.

### TiO_2_ Coating

TiO_2_ coating on the pre-cut panels for the UV reactor was carried out by an application protocol provided by PureTi. In addition, training was provided by SATA (Spring Valley, MN, USA) for accurate spraying control. The temperature (25°C) and relative humidity (40–45%) were adjusted to prevent instant evaporation of the sprayed TiO_2_ solution (nanostructured anatase 10 μg TiO_2_; PureTi) before application. After cleaning the surface of the experiment panel, TiO_2_ solution was sprayed. The spray pressure was adjusted to 60 psi with a regulator from the compressor, and the distance between the panel and the spray was ~0.15 m (6 in.) at an angle of 90 degrees. Coated panels were dried for 3 days.

### Experimental Fan Calibration

**Figure 2:**
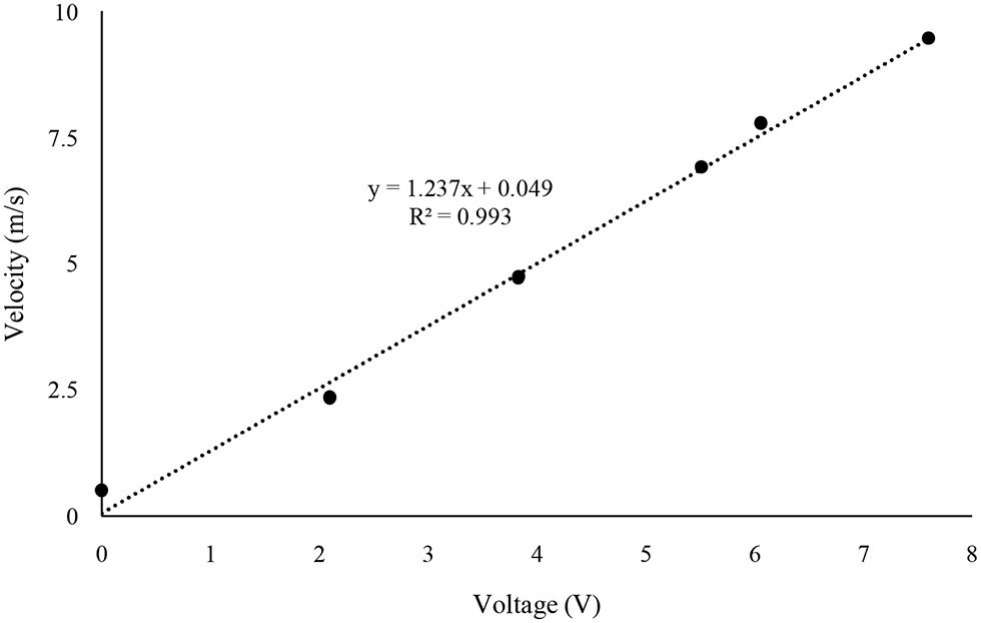
Calibration of air wind speed sensor.

The fan calibration was performed by measuring the velocity of each 0.012-m distance triplicated from the top of the fan and averaging a total of 8 points air velocity (Table 1). The dimmer switch was adjusted to measure the maximum, medium, and minimum air velocity of the pan. These three velocities were named as setting 1, 2, and 3 from the fastest speed order. In the experiments, fan velocity used settings 2.0, 2.5, and 3.0 to set typical air exchange rates inside mechanically ventilated barns. The treatment times in the inside of the reactor corresponding to the three air velocities are 40, 100, and 170 s, respectively. The air velocity of the fan was measured using a wind speed sensor (Modern Device, Providence, RI, USA) with Arduino Uno (Arduino LLC, Boston, MA, USA). The wind sensor was calibrated using the value of volts from the sensor at the WTM-1000 mini wind tunnel (Omega Engineering, Norwalk, CT, USA) from 0 to 10 m·s^−1^ (Figure 2).

**Table 1:** Calibration of air velocity.

**Velocity control setting**	**Distance from top of fan (m)**								**Average (m s^**−1**^)**
	**0.013**	**0.025**	**0.037**	**0.05**	**0.062**	**0.074**	**0.087**	**0.099**	
1.0	1.54	1.50	1.22	0.81	0.81	1.03	1.00	1.09	1.14 ± 0.31
2.0	1.27	1.21	0.92	0.45	0.49	0.55	0.64	0.60	0.71 ± 0.32
2.5	0.54	0.35	0.26	0.17	0.19	0.25	0.29	0.29	0.29 ± 0.12
3.0	0.30	0.23	0.21	0.11	0.10	0.13	0.16	0.17	0.18 ± 0.07

### UV-A Light Sources

**Figure 3:**
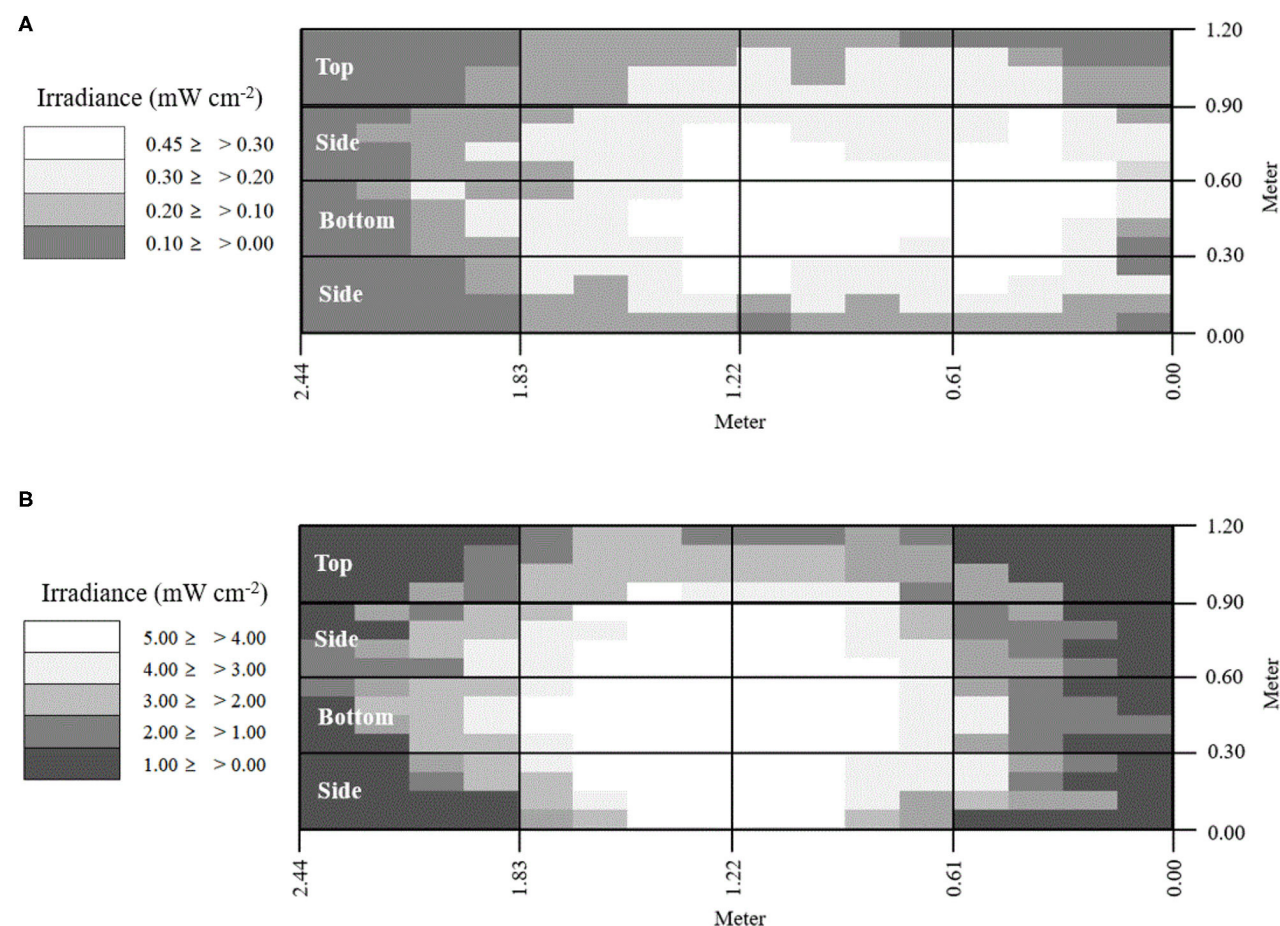
Surface map of UV-A light effective exposure. **(A)** Fluorescent, **(B)** LED.

Two fluorescent light bulbs were combined with one lamp, and a total of two lamps were used in this study. The LED used an aluminum board equipped with 108 LED chips. Light intensity was measured with an ILT-1700 radiometer (International Light Technologies, Peabody, MA, USA) equipped with an NS365 filter and SED033 detector (International Light Technologies, Peabody, MA, USA). For economic analysis, power consumption was measured using a wattage meter (P3, Lexington, NY, USA). The LED had ~10 times greater intensity and lower power consumption compared with the fluorescent lamp (Table 2 and Figure A1). The effective exposure of light intensity was mapped on the interior surfaces of the reactor (Figure 3).

**Table 2:** Comparison of experimental UV-A lamps.

	**Fluorescent**	**LED**
Total light intensity (mW·cm^−2^)	0.44	4.85
Power (W)	48.2	43.3
Lamp's position inside the reactor	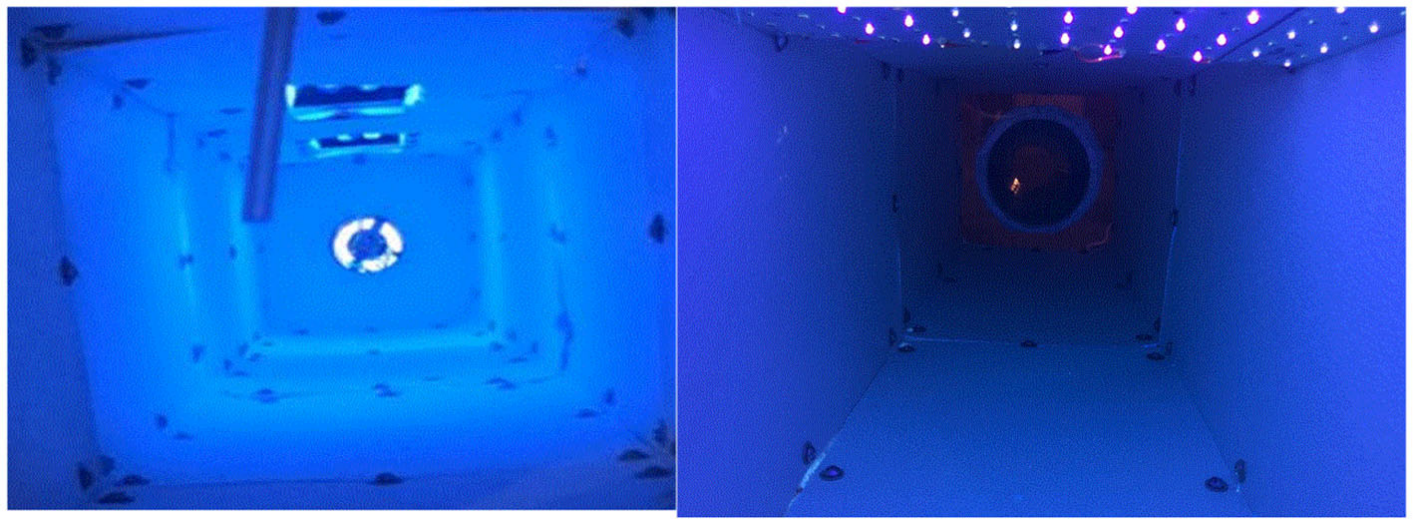	

### Teaching Poultry Farm

**Figure 4:**
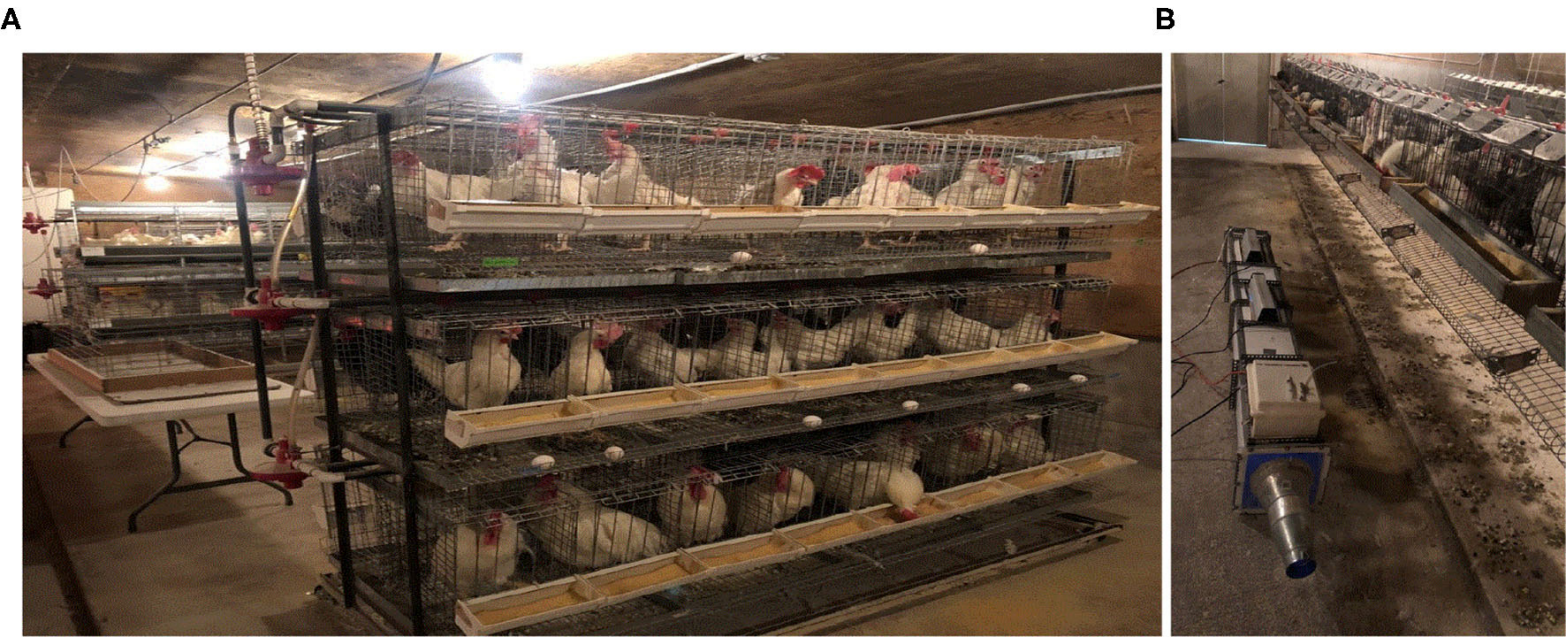
Pilot-scale mitigation was tested on a teaching poultry farm. **(A)** Interior of the teaching poultry barn, **(B)** UV-A reactor.

Pilot-scale testing was conducted at ISU Poultry Teaching Farm (Ames, IA, USA). The study was not using animals, and they were not exposed to UV-A. The performance of UV-A reactor was tested in realistic barn conditions where the environmental parameters (temperature, relative humidity, ventilation), dust, and gases were representative of the conditions inside poultry barns in general. The teaching farm is a caged facility with about 200 laying hens (Figure 4A). Animal density is 0.045–0.056 m^3^ head^−1^. Once a day, the manure was cleaned manually using a scraper. The teaching farm was set up with a side ventilation system in which the flow rate of the fan was automatically changed according to the temperature. The UV-A reactor was located in a nearby manure collector site (Figure 4B).

### Ammonia and Hydrogen Sulfide

A gas monitoring system (OMS-300; Smart Control & Sensing, Daejeon, Republic of Korea) equipped with electrochemical gas sensors of Membrapor (Wallisellen, Switzerland) was used to measure NH_3_ (NH3/CR-200) and H_2_S (H2S/C-50) concentrations. Both gas sensors were calibrated with standard gases before the experiment. The calibration result of both sensors was *R*^2^ > 0.98.

### Volatile Organic Compounds

Samples were collected and analyzed in the same method as in the previous study (Maurer and Koziel, 2019). Air samples for VOC measurements were collected using 1-L glass gas sampling bulbs (Supelco, Bellefonte, PA, USA). Air samples were taken using a portable vacuum sampling pump (Leland Legacy; SKC, Eighty-Four, PA, USA) with a set flow rate of 5 L min^−1^ for 1 min and analyzed in the same day. Chemical analyses of poultry odorants were completed using a solid-phase microextraction (SPME) (50/30 μm DVB/CAR/PDMS, 2-cm-long fibers; Supelco) using static extraction for 1 h at room temperature and gas chromatography–mass spectrometry (GC-MS) system for analyses (Agilent 6890 GC; Microanalytics, Round Rock, TX, USA).

### Greenhouse Gases

Greenhouse gases (GHGs) methane (CH_4_), carbon dioxide (CO_2_), and nitrous oxide (N_2_O) were measured. GHG samples were collected using syringes and 5.9-ml Exetainer vials (Labco Limited, UK) and were analyzed for GHG concentrations on a GC equipped with flame ionization and electron capture detectors (SRI Instruments, Torrance, CA, USA). Samples were analyzed on the day of collection. Standard calibrations were constructed daily using 10.3 and 20.5 ppm CH_4_, 1,005 and 4,010 ppm CO_2_, and 0.101 ppm and 1.01 ppm N_2_O, and pure helium used to 0 ppm (Air Liquide America, Plumsteadville, PA, USA).

### Ozone

The O_3_ detector was connected to the monitoring system (Series 500 monitor; Aeroqual, New Zealand) and installed at the sampling site inside the reactor. The ozone sensor (OZS, Aeroqual) was sent to the professional company (Gas Sensing, IA, USA) for certified calibration before use. The detection range was from 0 to 50 ppb.

### Odor

Odor samples were collected from the incoming and outgoing air sampling ports of the UV reactor in 10-L Tedlar sample bags using a Vac-U-Chamber (SKC) and sampling pump (SKC). Tedlar sampling bags were pre-cleaned by flushing with clean air three times before use. Odor samples were analyzed using a dynamic triangular forced-choice olfactometer (St. Croix Sensory, Stillwater, MN, USA). Four trained panelists at two repetitions each were used in the analysis of each sample. Each sample was presented to the panelists from low concentration to higher concentrations; each dilution level doubled the concentration of the odor.

### Data Measurement and Analysis

Gas samples were collected after 30 min of equilibration time under each treatment condition. The overall mean percent reduction (mitigation) for each measured gas was estimated using:

(1) $$\begin{array}{r} \%\;Reduction=\frac{E_{con}-\;E_{Treat}}{E_{con}}\;\times \;100 \end{array}$$

where E_Con_ and E_Treat_ are the mean measured concentrations in control and treated air, respectively.

Emission rates were calculated as a product of measured gas concentrations and the total airflow rate through the wind tunnel, adjusted for standard conditions and dry air using collected environmental data. The overall mean mitigation of each measured gas emission was estimated using

(2) $$\begin{array}{l} Mitigation\;of\;E\;\left(g\cdot \min^{-1}\right)=\\ \left(C_{con}\times V\times \frac{273.15\times MW}{\left(K_{Con}\right)\times 2.24\times 10^{4}}-C_{Treat}\times V\times \frac{273.15\times MW}{\left(K_{Treat}\right)\times 2.24\times 10^{4}}\right) \end{array}$$

where mitigation of E (g min^−1^) is the mitigation of gas emission. C_Con_ and C_Treat_ are the mean measured concentrations in control and treated air (mL m^−3^), respectively. V is the ventilation rate (m^3^ min^−1^). MW is the molecular weight of target gas (g mol^−1^). K_Con_ and K_Treat_ are the temperature in control and treated air (K), respectively. The 2.24 × 10^4^ is an ideal gas conversion factor for liters to moles at 273.15 K.

The electric power consumption was calculated as the measured power at each UV-A light source. Electric power consumption (kWh) was calculated using Equation 3.

(3) $$\begin{array}{r} Electric\;power\;consumption\;\left(kWh\right)=P\;\times \;T_{s}\\ \text{*****************}\times \;{3,600}^{-1}\;\times \;10^{-3} \end{array}$$

where P is the power (W) and T_s_ is the treatment time (s).

Energy efficiency was calculated using the power consumption per mitigation of gas emission at treatment time (Equation 4), and the power consumption was changed as cost. The cost ($) was calculated by converting the power consumption (kWh) into dollars (12 cents is 1 kWh).

(4) $$\begin{array}{r} Energy\;efficiency\;\left(kWh{\cdot g}^{-1}\cdot min\right)\;=\\ \text{*************}\frac{The\;electric\;power\;consumption\;\left(kWh\right)}{Reduction\;of\;E\;\left(g\cdot \overset{-1}{\min}\right)\;} \end{array}$$

### Statistical Analysis

The program of R (version 3.6.2) was used to analyze the mitigation effect of UV-A irradiative treatment for target gases on the poultry farm. The parameters of catalyst, lamp type, and treatment time between control concentration and treatment concentration were statistically analyzed using one-way ANOVA. The statistical difference was confirmed by obtaining the *p*-value through the paired Tukey test. A significant difference was defined for a *p* < 0.05 in this study.

## Results

### Environmental Parameters

The average temperature inside the poultry barn was 25 ± 3°C, and the average RH was 59 ± 4%. The average temperature of the gas after the fluorescent UV-A light irradiation and LED was 27 ± 2 and 28 ± 3°C, respectively. In addition, RH decreased by about 3% after UV irradiation. Therefore, after the UV-A light irradiation, the temperature was increased, but RH was decreased. It was confirmed that the concentration of target gases was significantly reduced because of the increased ventilation in the farm from 12:00 (e.g., NH_3_ concentration is 10 ± 1 ppm at 12:00). Therefore, all samples were collected before 10:00.

### Ammonia and Hydrogen Sulfide

The average NH_3_ concentration in the poultry barn without treatment was 23±3 ppm (Table A1). In the case of photolysis (Table 3 and Figure A2), there was no statistically significant NH_3_ mitigation in all experimental conditions (*p* > 0.05). For photocatalysis (Figure A4 and Table A4), the percent NH_3_ reduction was 5% when using fluorescent UV-A and 9% when using LED UV-A (*p* < 0.05). However, there was no significant mitigation difference between treatment time of 100 s and 170 s (*p* > 0.05) when using the LED source. The H_2_S concentrations were too low for measurement with the instrumentation used (0–5 ppb). Thus, reductions could not be estimated.

**Table 3:** Summary of NH_3_ mitigation with UV-A treatment.

**Treatment time (s)**	**Photolysis**		**Photocatalysis**	
	**Fluorescent**	**LED**	**Fluorescent**	**LED**
40	1.7 (0.35)	0.1 (0.95)	−0.2 (0.89)	2.5 (0.07)
100	1.1 (0.29)	0.9 (0.22)	2.6 (0.07)	**8.1 (0.04)**
170	0.7 (0.23)	1.2 (0.25)	**5.2 (0.04)**	**8.7 (0.01)**

*Values in table report % reduction (p-value). Bold font means a statistical difference (p < 0.05)*.

### Volatile Organic Compounds

In the case of photolysis (Table 4), three VOCs showed statistically significant percent reductions and were limited to the longest treatment time (170 s), i.e., dimethyl disulfide (25.8% under fluorescent UV-A), *p*-cresol (35.6%), and indole (31.4%) under LED UV-A. In the case of photocatalysis, five VOCs showed significant percent reductions, i.e., *p*-cresol (32.2% under fluorescent UV-A), and dimethyl disulfide, butanoic acid, *p*-cresol, indole, and skatole showed 31.2–61.9% reductions under LED UV-A. In general, treatment with the LED at the longest treatment time (170 s) was most effective in reducing the concentrations of most VOCs measured.

**Table 4:** Mitigation of VOCs.

	**Fluorescent**						**LED**					
	**Treatment time (s)**											
	**40**		**100**		**170**		**40**		**100**		**170**	
**Target compound**	**Photo lysis**	**Photo catalysis**	**Photo lysis**	**Photo catalysis**	**Photo lysis**	**Photo catalysis**	**Photo lysis**	**Photo catalysis**	**Photo lysis**	**Photo catalysis**	**Photo lysis**	**Photo catalysis**
DMDS	16 (0.62)	16 (0.69)	−24 (0.66)	22(0.30)	34 (0.20)	26 (0.54)	21 (0.13)	38 (0.37)	18 (0.14)	33 (0.26)	40 (0.06)	35 (0.30)
DEDS	14 (0.26)	23 (0.26)	−2 (0.90)	13 (0.67)	**26 (0.03)**	19 (0.06)	−9 (0.38)	18 (0.66)	14 (0.28)	26 (0.08)	19 (0.51)	**47 (0.02)**
DMTS	−7 (0.81)	18 (0.27)	11 (0.82)	52 (0.05)	30 (0.21)	36 (0.34)	−64 (0.42)	1 (0.99)	10 (0.36)	8 (0.51)	−16 (0.41)	43 (0.08)
AA	26 (0.69)	−46 (0.68)	−10 (0.68)	39 (0.68)	26 (0.62)	59 (0.23)	27 (0.52)	−44 (0.16)	0 (0.99)	44 (0.37)	−17 (0.49)	75 (0.42)
PA	−47 (0.56)	29 (0.59)	1.0 (0.99)	33 (0.54)	−8 (0.88)	51 (0.09)	−53 (0.40)	−5 (0.92)	−2 (0.97)	4 (0.92)	−14 (0.23)	36 (0.47)
IA	1 (0.95)	20 (0.76)	−33 (0.39)	−21 (0.82)	10 (0.74)	−16 (0.44)	6 (0.56)	6 (0.76)	−6 (0.87)	−44 (0.53)	−21 (0.23)	29 (0.53)
BA	−51 (0.50)	28 (0.06)	−38 (0.61)	6 (0.87)	27 (0.27)	22 (0.71)	17 (0.75)	22 (0.11)	−7 (0.88)	16 (0.79)	−4 (0.57)	**62 (<0.01)**
IVA	−33 (0.70)	−21(0.20)	−14 (0.84)	34 (0.09)	−19 (0.57)	38 (0.30)	−36 (0.66)	–40 (0.64)	−44 (0.14)	23 (0.70)	−49 (0.07)	40 (0.41)
Phenol	−42 (0.69)	−25 (0.39)	−39 (0.50)	−37 (0.61)	33 (0.14)	−3 (0.92)	−22 (0.57)	−5 (0.84)	16 (0.11)	−3 (0.94)	28 (0.31)	−27 (0.63)
*p*-Cresol	3 (0.96)	31 (0.56)	23 (0.79)	**32 (0.04)**	26 (0.26)	11 (0.90)	5 (0.64)	22 (0.64)	8 (0.73)	52 (0.04)	**36 (0.01)**	**49 (0.03)**
Indole	4 (0.85)	−6 (0.90)	5 (0.95)	45(0.17)	19 (0.59)	54 (0.11)	0 (0.98)	15 (0.23)	12 (0.27)	**31 (0.03)**	**31 (0.02)**	21 (0.69)
Skatole	−3 (0.91)	47 (0.06)	−34 (0.83)	−36 (0.42)	3 (0.92)	19 (0.80)	10 (0.78)	54 (0.18)	8 (0.85)	12 (0.90)	−7 (0.93)	**35 (0.04)**

*Dimethyl disulfide (DMDS), diethyl disulfide (DEDS), dimethyl trisulfide (DMTS), acetic acid (AA), propanoic acid (PA), isopentanoic acid (IA), butanoic acid (BA), isovaleric acid (IVA). Values in table report % reduction (p-values), bold font signifies statistical significance*.

### Greenhouse Gases

The average GHGs concentration in the poultry barn was 2.5 ± 0.2 (CH_4_), 465 ± 48 (CO_2_), and 0.28 ± 0.03 (Table A2, N_2_O) ppm. For CH_4_ and CO_2_, there was no statistically different change in their concentration (Table 5). However, N_2_O showed under photocatalysis an average 6% reduction with fluorescent UV-A and 9% with LED UV-A. In addition, The photolysis using an only LED UV light source still showed a 7% reduction. The N_2_O mitigation showed a higher percent reduction with the high light intensity and with longer treatment time (*p* < 0.05).

**Table 5:** Comparison of greenhouse gas mitigation.

**T time^a^ (s)**	**CH**_****4****_				**CO**_****2****_				**N**_****2****_**O**			
	**Photolysis**		**Photocatalysis**		**Photolysis**		**Photocatalysis**		**Photolysis**		**Photocatalysis**	
	**Flu^b^**	**LED**	**Flu**	**LED**	**Flu**	**LED**	**Flu**	**LED**	**Flu**	**LED**	**Flu**	**LED**
40	−3.9 (0.61)	−2.2 (0.56)	2.4 (0.15)	−4.3 (0.07)	7.4 (0.15)	−1.4 (0.65)	2.3 (0.12)	5.3 (0.28)	2.2 (0.21)	3.8 (0.31)	1.2 (0.45)	5.3 (0.06)
100	−0.6 (0.31)	−6.8 (0.14)	−2.5 (0.28)	2.6 (0.31)	1.9 (0.09)	−2.7 (0.22)	−0.2 (0.55)	1.8 (0.42)	2.3 (0.35)	2.6 (0.42)	**5.6 (0.04)**	**6.9 (0.02)**
170	−1.7 (0.09)	0.3 (0.93)	3.8 (0.25)	1.7 (0.48)	0.4 (0.88)	11.8 (0.32)	−1.2 (0.58)	7.3 (0.05)	1.2 (0.32)	**7.3 (0.02)**	**7.5 (0.01)**	**12.5 (0.01)**

a *Treatment time*;

b *Fluorescent*.

*Values in table report % reduction (p-value). Bold font means a statistical difference (p < 0.05)*.

### Odor

Except for one of 12 treatments, the odor mitigation was represented (Table 6). Odor percent reduction was statistically different only in long treatment time and high light intensity conditions (LED, 170 s). The odor percent reduction was presented at 18% (p < 0.05). The odor unit (OU_e_·m^−3^) decreased from 582 ± 25 to 475 ± 38 in condition with statistical differences (Table A3).

**Table 6:** Mitigation of odor.

**Treatment time (s)**	**Odor**			
	**Photolysis**		**Photocatalysis**	
	**Fluorescent**	**LED**	**Fluorescent**	**LED**
40	0.1 (0.98)	2.2 (0.52)	6.8 (0.43)	15.7 (0.22)
100	15.0 (0.31)	−21.9 (0.14)	9.3 (0.15)	7.3 (0.25)
170	4.9 (0.59)	9.6(0.64)	7.6 (0.38)	**18.4 (0.01)**

*Values in table report % reduction (p-value). Bold font means a statistical difference (p < 0.05)*.

### Ozone

The average O_3_ concentration in control samples was 9.0±4.7 ppb (Table 7). The average O_3_ concentration after treatments was 0.3 ± 1.2 (ppb). Both photolysis and photocatalysis showed a similar tendency to decrease O_3_ concentration (Figures A3,A5 and Table A5). The LED-based irradiations showed a greater reduction than fluorescent (100 vs. 87%). However, there was no statistically significant difference between them (*p* > 0.05).

**Table 7:** Mitigation of ozone concentration (ppb).

**Treatment time (s)**	**UV type**	**Photolysis**		**Photocatalysis**	
		**Control**	**Treatment**	**Control**	**Treatment**
40	Fluorescent	10.0 ± 2.7	0.0 ± 0.0	1.3 ± 1.5	0.0 ± 0.0
	LED	11.3 ± 1.5	0.0 ± 0.0	7.7 ± 1.2	0.0 ± 0.0
100	Fluorescent	17.3 ± 2.5	2.3 ± 4.0	6.7 ± 1.5	0.0 ± 0.0
	LED	8.7 ± 1.2	0.0 ± 0.0	2.7 ± 1.5	0.0 ± 0.0
170	Fluorescent	6.3 ± 1.5	0.7 ± 1.2	13.3 ± 2.1	0.0 ± 0.0
	LED	12.3 ± 1.2	0.0 ± 0.0	5.3 ± 1.2	0.0 ± 0.0

*Values in table report average concentration ± standard deviation. All figures are statistically significant*.

## Discussion

### Comparison With Laboratory-Scale and Pilot-Scale Experiments

This pilot-scale study showed the percent reduction in the concentrations of NH_3_, N_2_O, O_3_, and some types of VOCs in poultry barn exhaust as a result of direct and indirect photolytic treatment. The results show similar trends as the laboratory experiment that used the same UV-A light source and TiO_2_ density. A comparison of the laboratory and pilot scales are summarized in Table 8. On the laboratory scale (Lee et al., 2020), an RH of 12% was reported as the optimal humidity condition for reducing target gases. As the treatment time decreased, the light intensity decreased, the relative humidity increased, and dust accumulation increased, the percent reduction gradually decreased. As a representative example, when the RH increased from 12 to 60%, the percent reduction of NH_3_ declined from 19 to 6%. Therefore, the mitigation for the target gas was expected to be low, considering the actual RH and dust inside the poultry farm.

**Table 8:** Percent reduction of target gases with UV-A photocatalysis in the poultry farm condition.

**References**	**Experiment conditions**	**Average % reduction of target gases**					
		**NH_**3**_**	**CO_**2**_**	**N_**2**_O**	**O_**3**_**	**VOCs**	**Odor**
Lee et al. (2020)	Laboratory scale Temp^a^: 25 ± 3°C RH^b^: 12% T time^c^ (s): 200	**18.7**	**3.8**	**9.5**	**48.4**	Not investigated	Not investigated
	Laboratory scale Temp^a^: 25 ± 3°C RH^b^: 60% T time^c^ (s): 40	5.1	1.1	5.2	27.6	Not investigated	Not investigated
	Laboratory scale Temp^a^: 25 ± 3°C RH^b^: 60% T time^c^ (s): 200	**6.2**	9.3	**4.9**	**37.5**	Not investigated	Not investigated
This study	Pilot scale Temp^a^: 28 ± 3°C RH^b^: 56% T time^c^ (s): 40	2.5	5.3	5.3	**100**	No % reduction	15.7
	Pilot scale Temp^a^: 28 ± 3°C RH^b^: 56% T time^c^ (s): 100	**8.1**	1.8	**6.9**	**100**	**31.4 (Indole)**	7.3
	Pilot scale Temp^a^: 28 ± 3°C RH^b^: 56% T time^c^ (s): 170	**8.7**	7.3	**12.5**	**100**	**47.2 (DEDS) 61.9 (BA) 49.3 (*****p*****-Cresol) 35.4 (Skatole)**	**18.4**

a *Temperature*;

b *Relative humidity*;

c *Treatment time; diethyl disulfide (DEDS), butanoic acid (BA); bold font signifies a statistical difference (p < 0.05)*.

However, unlike these concerns, there was a statistically significant decrease in NH_3_, N_2_O, and O_3_. In the case of NH_3_ concentration, the percent reduction increased with the increase of the light intensity. However, it did not show a statistical difference with the treatment time (100 vs. 170 s). For N_2_O concentration, the percent reduction improved as the light intensity, and the treatment time increased. O_3_ was detected in very low concentrations in the poultry farm; then, it was almost mitigated after UV irradiation. Interestingly, CO_2_ did not show a percent reduction effect, which is a different result compared with that from the laboratory scale. It is considered as a result reflecting that there is no chemical reason for the reduction of CO_2_ under general photocatalysis conditions with normal TiO_2_. Pilot studies also showed that photocatalysis has the effect of improving indoor air quality by reducing some types of VOC and odor. Therefore, the application of photocatalysis in the poultry farm through laboratory-scale and pilot-scale results is considered to be a potential and positive technology in terms of reducing odorous gases, decreasing GHGs, and improving indoor air quality.

### Economic Analysis

The advent of inexpensive LED lighting has reduced the cost per photon significantly below what it was with previous fluorescent lamp technology. Clearly, there remains a direct relationship between treatment time and energy cost, however. Here, we consider the cost of reducing 1 g of the target gas, considering only statistically significant reduction results. If NH_3_ is the target gas, the cost was found to be $0.7 to $3.6 to remove 1 g of material per minute (Table 9) depending on the light intensity. A cost of $32.1–$79.0 was estimated to remove 1 g of N_2_O per minute (Table 10), depending on the light intensity. For NH_3_ and N_2_O, however, removal is not predicted with statistical significance, for periods of high ventilation rates, such as summer, i.e., when treatment times are short (i.e., 40 s in the current apparatus). It is likely that increased light intensity or methods that would increase contact (treatment) time in the barn (with correspondingly higher energy costs) could be made effective. Importantly, the estimated cost is considered reasonably expensive compared with other odor treatment techniques and equipment presented in previous studies (Koziel et al., 2008; Maurer et al., 2017). In terms of cost, shorter treatment time is better (100 s) (Tables 9, 10). However, it is longer treatment time (170 s), which results in a percent reduction for more target gases (VOCs and odor, Table 8), that would be considered more suitable for farm application.

**Table 9:** Economic analysis of NH_3_.

	**Fluorescent**			**LED**		
**Treatment time (s)**	**40**	**100**	**170**	**40**	**100**	**170**
Reduction rate (%)	−0.2 ± 2.4	2.6 ± 1.2	**5.2** **±** **1.7**	2.5 ± 1.2	**8.1** **±** **3.2**	**8.7** **±** **1.0**
Reduction of NH_3_ emission (μg·min^−1^)	15.7 ± 109.9	59.8 ± 22.9	81.3 ± 26.1	171.1 ± 62.8	229.1 ± 77.0	134.5 ± 22.6
Energy efficiency (kWh·g^−1^·min)	3.2 ± 7.2	25.2 ± 11.4	30.4 ± 11.3	3.0 ± 1.0	5.8 ± 2.4	15.5 ± 2.4
Cost per reduction of NH_3_ emission (dollar·g^−1^·min)	0.4 ± 0.9	3.0 ± 1.4	3.6 ± 1.4	0.4 ± 0.1	0.7 ± 0.3	1.9 ± 0.3

Bold font signifies a statistical difference (p < 0.05).

**Table 10:** Economic analysis of N_2_O.

	**Fluorescent**			**LED**		
**Treatment time (s)**	**40**	**100**	**170**	**40**	**100**	**170**
Reduction rate (%)	1.2 ± 2.3	**5.5** **±** **1.9**	**7.5** **±** **1.5**	5.3 ± 2.2	**6.9** **±** **1.5**	**12.4** **±** **2.1**
Reduction of N_2_O emission (μg·min^−1^)	2.1 ± 3.8	4.1 ± 1.4	3.6 ± 0.7	8.6 ± 3.8	4.6 ± 1.0	5.1 ± 0.9
Energy efficiency (kWh·g^−1^·min)	17 ± 287	362 ± 150	658 ± 137	66 ± 34	268 ± 62	407 ± 70
Cost per reduction of N_2_O emission (dollar·g^−1^·min)	2.0 ± 34.5	43.5 ± 18.0	79.0 ± 16.5	7.9 ± 4.0	32.1 ± 7.4	48.8 ± 8.4

*Bold font signifies a statistical difference (p < 0.05)*.

In 170 s of treatment time with LED lighting, we estimate that ~548 mg of NH_3_, ~21 mg of N_2_O, and ~3.3 mg of O_3_ could be removed from the atmosphere for the cost of $1 per minute (Table 11). For reference, the NH_3_ emissions in a poultry barn can be ~1 g NH_3_ bird^−1^ day^−1^ (Wheeler et al., 2003; Xin et al., 2003) or ~0.7 mg NH_3_ bird^−1^ min^−1^. UV-A technology with 170 s treatment time can remove 66 mg of NH_3_ per minute while consuming 1 kWh of electricity (Table 11), an equivalent of 100% mitigation of NH_3_ emissions from ~100 birds. Therefore, NH_3_ emitted from the farm with ~100 birds can be removed by changing the light bulb installed on the farm to UV-A light. However, research on reducing the lighting cost while reducing emissions by replacing the ordinary lights with LED UV-A lights is warranted.

**Table 11:** Economic analysis when using photocatalysis with 170 s treatment time under LED.

**Target gases**	**NH_**3**_**	**N_**2**_O**	**O_**3**_**
Reduction rate (%)	8.7 ± 1.0	12.4 ± 2.1	100 ± 0.0
Reduction of target gases emission (μg·min^−1^)	135 ± 22.6	5.1 ± 0.9	0.8 ± 0.2
Energy efficiency of mitigating target gases (kWh·g^−1^·min)	15.5 ± 2.4	407 ± 70.2	2,610 ± 645
Reduction of gas emission·power consumption (mg·min^−1^·kWh^−1^)	65.8 ± 11.0	2.5 ± 0.5	0.4 ± 0.1
Reduction of gas emission per one dollar (mg·min^−1^·dollar^−1^)	548 ± 91.9	20.9 ± 3.8	3.3 ± 0.7

### Evaluation of Photocatalysis With UV-A Light Based on TiO_2_ in the Livestock Environment

Table 8 summarizes previous research on the mitigation of selected target gases via photocatalysis with UV-A in livestock-relevant environmental conditions. The combined results show that photolysis with UV-A with assistance by TiO_2_ coatings yields significant reductions of some target gases. However, in all instances of Table 12, mitigation is more effective with the use of TiO_2_ coatings and photocatalytic assistance.

**Table 12:** Summary of % reduction for selected target gas with TiO_2_ and UV-A light in the livestock farm condition.

**Reference**	**Experiment conditions**	**Coating material**	**UV type (wavelength)**	**Light intensity**	**Target gas (% ave^a^ reduction)**	
					**Photolysis**	**Photocatalysis**
Yao and Feilberg (2015)	Simulated livestock facility (laboratory) Tem^b^: Not reported RH^c^: 50% T time^d^ (s): 0.24	TiO_2_ (1.5 m^2^·g^−1^)	UV-A (315–400 nm) Main: 368 nm	2.3–5.6 mW·cm^−2^	Not reported	**H**_**2**_**S (4.2–14.0) MT (80.1–87.4) DMS (92.2–95.8) DMDS (82.5–90.8) 1-Butanol (92.6–95.1) AA (81.0–88.5) PA (96.5–97.8) BA (98.4–99.2) VA (98.8–99.0)**
Costa et al. (2012)	Swine farm (farm) Tem^b^: 26°C RH^c^: 56% T time^d^ (s): 72	TiO_2_ (7 mg·cm^2^)	UV-A (315–400 nm)	0.05–0.45 lux	Not reported	NH_3_ (2.0) **CH**_**4**_ **(27.4)** CO_2_ (−4.5) N_2_O (−0.8) **PM**_**10**_ **(17.0)** PM_2.5_ (−8.1)
Guarino et al. (2008)	Swine farm (farm) Tem^b^: 24°C RH^c^: 54% T time^d^ (s): 363	TiO_2_ (7 mg·cm^2^)	UV-A (315–400 nm)	Not reported	Not reported	**NH**_**3**_ **(30.5) CH**_**4**_ **(10.8) CO**_**2**_ **(15.3)** N_2_O (4.2)
Zhu et al. (2017)	Simulated swine farm (laboratory) Tem^b^: 40°C RH^c^: 40% T time^d^ (s): 40, 200	TiO_2_ (10 μg·cm^2^)	UV-A (365 nm)	0.06 mW·cm^−2^	Not reported	**DMDS (35.0, 40.4) DEDS (27.7, 81.0) DMTS (37.1, 76.3) BA (62.2, 86.9) Guaiacol (37.4, 100.0)** ***p*****-Cresol (27.4, 93.8)**
(Maurer and Koziel, 2019)	Swine farm (pilot) Tem^a^: 22–26°C RH^c^: 36–80% T time^d^ (s): 24, 47	TiO_2_ (10 μg·cm^2^)	UV-A (365 nm)	<0.04 mW·cm^−2^	**N**_**2**_**O (4.2, 7.6)**	CH_4_ (−1.4, −2.2) **CO**_**2**_ **(**0.0, **−3.1) N**_**2**_**O (7.3, 8.7) AA (−52.9**, −19.7**) Odor (**not reported, **16.3)** ***p*****-Cresol (**−21.4, **22.0)**
(Lee et al., 2020)	Simulated poultry farm (laboratory) Tem^b^: 25 ± 3°C RH^c^: 12% T time^d^ (s): 40, 200	TiO_2_ (10 μg·cm^2^)	UV-A (365 nm)	4.85 mW·cm^−2^	NH_3_ (0.0, 0.0) CO_2_ (4.0, 2.3) **N**_**2**_**O (**3.3, **6.5) O**_**3**_ **(**26.0, **12.9)**	**NH**_**3**_ **(10.4, 18.7) CO**_**2**_ **(**4.2, **3.8) N**_**2**_**O (**8.9, **9.5) O**_**3**_ **(37.0, 48.4)**
This study	Poultry farm (pilot) Tem^b^: 28 ± 3°C RH^c^: 56% T time^d^ (s): 40, 170	TiO_2_ (10 μg·cm^2^)	UV-A (365 nm)	4.85 mW·cm^−2^	NH_3_ (0.1, 1.2) CO_2_ (−1.4, 11.8) **N**_**2**_**O (**3.8, **7.3) O**_**3**_ **(100, 100)** ***p*****-Cresol (**5.2, **35.6) Indole (**0.3, **31.4)**	**NH**_**3**_ **(**2.5**, 8.7)** CO_2_ (5.3, 7.3) **N**_**2**_**O (**5.3, **12.5) O**_**3**_ **(100, 100)Odor (**15.7, **18.4) DEDS (**18.1, **47.2) BA (**22.1, **61.9)** ***p*****-Cresol (**21.8, **49.3) Skatole (**53.6, **35.4)**

a *Mean*;

b *Temperature*;

c *Relative humidity*;

d *Treatment time; methanethiol (MT), dimethyl sulfide (DMS), dimethyl disulfide (DMDS), diethyl disulfide (DEDS), dimethyl trisulfide (DMTS), acetic Acid (AA), propionic acid (PA), butanoic acid (BA), valeric acid (VA). Bold font signifies a statistical difference (p < 0.05)*.

The combination of UV-A with TiO_2_ showed a reduction in NH_3_, H_2_S, GHGs, O_3_, VOCs, and odor. NH_3_ reduced about 9–31% depending on the light intensity, the treatment time, and the coating density, whereas H_2_S showed a 4–14% reduction depending on light intensity. In the case of NH_3_, the mitigation was reported to be 31% at the farm scale, whereas at the laboratory scale and pilot scale it showed 10 to 20%. The effect of NH_3_ reduction is difficult to directly compare in both of the previous researches because of the difference of TiO_2_ coating thickness and light intensity. However, in the farm-scale study, the NH_3_ concentration in the farm (1.9 ppb) is lower than the general NH_3_ concentration in the swine farm. Therefore, further research is needed. H_2_S showed a reduction effect in the experiment of the laboratory scale using a standard gas of 1,000 ppm as a control. However, the exact reduction could not be determined in the pilot-scale and the other laboratory-scale study because of a low concentration (below 5 ppm).

For the GHGs, the previous laboratory-scale study did not find a significant reduction in CH_4_ using photocatalysis, despite the well-known oxidation of hydrocarbons under other photocatalytic conditions. However, here, moderate reductions (11–27%) were observed with the larger scale and high coating thickness. A surprising slight decrease in CO_2_ was observed in the laboratory-scale and farm-scale experiments, but none is reported here. In general, CO_2_ is the oxidative endpoint for photocatalytic oxidation of virtually all carbon-containing compounds under conditions like those used here, and thus its mitigation would not derive from its chemical removal. N_2_O was found to decrease by 7–13% in the laboratory and pilot scales, but no statistically significant reduction was reported in the farm-scale experiments.

In the case of O_3_, the concentration decreases during the UV-A irradiation, which was shown in this study and previous studies on a laboratory scale. The concentration of O_3_ showed more reduction at the pilot scale (100%) than at the laboratory scale (48%). It is considered that the O_3_ concentration is further reduced to decompose the target gases because more target gases and malodorous organic compounds are present in the farm.

VOCs have been reported to reduce some types of organosulfur compounds, volatile fatty acids, phenols, and alcohol with photocatalysis. In the simulated livestock environment, it was reported that percent reduction was showed in methanethiol, dimethyl sulfide, dimethyl disulfide, and dimethyl trisulfide of organosulfur compounds, in acetic acid, propionic acid, butanoic acid, and valeric acid of volatile fatty acids, in *p*-cresol and guaiacol of phenols, and in 1-butanol. In the case of the poultry environment, the percent reduction was reported in diethyl disulfide of organosulfur compounds, in butanoic acid of volatile fatty acids, in *p*-cresol of phenol, and in indole and skatole, respectively. Odor mitigation was reported to decrease by 16–18%, reflecting the mitigation of VOCs and other target gas.

However, some people have a skeptical view of photocatalysis because of the by-products after photocatalysis. Through the results of previous studies (Table 13), some hazardous by-products such as N_2_O, ${\text{SO}}_{4}^{2-}$ (sulfate), CH_3_OH (methanol), methane by-products (C_2_H_6_, C_2_H_4_, CH_2_O_2_), and HCHO (formaldehyde) were reported. However, H_2_S, CH_4_, and CO_2_, which produce harmful by-products, did not show statistical mitigation in this study. In addition, no research has been conducted to investigate the by-product concentrations observed in the poultry farm after photocatalysis. It is assumed that the target gases of concern here would be at low concentrations, and the generated by-products will not likely be generated at harmful levels. Further research and scaling up to poultry barn applications are needed. Therefore, although further studies on the by-product and economic suitability are needed, the potential for photocatalytic technology with UV-A in the livestock farm is still favorably considered because of the mitigation of NH_3_, GHGs, O_3_, VOCs, and odor.

**Table 13:** List of major by-products after photocatalysis.

**References**	**Target gases**	**Major by-products after photocatalysis**
Jayanty et al. (1976), Levine and Calvert (1977), Mozzanega et al. (1979), Cant and Cole (1992)	NH_3_	N_2_O and N_2_
Canela et al. (1998), Portela et al. (2007, 2008, 2010), Alonso-Tellez et al. (2012)	H_2_S	SO_2_ and ${\text{SO}}_{4}^{2-}$
Taylor (2003), Yuliati and Yoshida (2008), Chen et al. (2016)	CH_4_	CH_3_OH, H, C_2_H_6_, C_2_H_4_, CH_2_O_2_, and CO_2_
Tan et al. (2006), Paramasivam et al. (2012)	CO_2_	CH_4_, CH_3_OH, HCHO, and CO
Blyholder and Tanaka (1971), Obalová et al. (2013)	N_2_O	N_2_ and O_2_
Lin et al. (2002)	O_3_	O_2_ and O
Koziel et al. (2008)	VOCs	Partially oxidized species, CO_2_, and H_2_O

## Conclusion

The results of the study provide evidence that a photocatalyst using TiO_2_ coating and UV-A light can reduce the target gas concentrations in poultry farm conditions. The photocatalysis reduced NH_3_, N_2_O, O_3_, VOCs, and odor. However, it did not affect H_2_S, CH_4_, and CO_2_. In the case of NH_3_ concentration, the percent reduction ranged from 5 to 9% and was affected by light intensity. However, there was no statistical increase with increasing treatment time. The percent reduction in N_2_O and O_3_ concentrations increased with increasing light intensity and treatment time (6–12% for N_2_O and 87–100% for O_3_). For VOCs, greater light intensity (LED) and longer treatment time (170 s) improved mitigation. The percent reduction was observed for DEDS (26–47%), BA (62%), *p*-cresol (32–49%), indole (31%), and skatole (35%) concentrations. The odor showed a statistical reduction of 18% only under LED with170 s treatment time. Preliminary economic analysis showed that the cost of removing 1 g of target gas per minute was $1.9 ± 0.3 for NH_3_, $48.8 ± 8.4 for N_2_O, and $312.8 ± 77.4 for O_3_ in the most effective conditions (LED with 170 s treatment time). The application of photocatalysis based on TiO_2_ with UV-A in the poultry farm is therefore considered to be potentially beneficial in terms of reducing odorous gases, decreasing GHGs, removing O_3_, and improving indoor air quality. Further research needs to be extended to farm-scale trials for investigating more detailed economic analysis and mitigation of target gases. In addition, it is also necessary to investigate the by-products after photocatalysis treatment for safe technology applications.

## Author Contributions

JK and WJ: conceptualization. JK: methodology. ML, JW, HA, and JK: validation. ML: formal analysis and writing—original draft preparation. ML, PL, BC, ZM, and CB: investigation. PL, BC, ZM, CB, and JK: resources. ML and JK: data curation. ML, HA, JK, and WJ: writing—review and editing. ML: visualization. HA and JK: supervision. JK and WJ: project administration and funding acquisition. All authors contributed to the article and approved the submitted version.

## Conflict of Interest

The authors declare that the research was conducted in the absence of any commercial or financial relationships that could be construed as a potential conflict of interest.
