# Supplementary material for: Pilot-Scale Testing of UV-A Light Treatment for Mitigation of NH3, H2S, GHGs, VOCs, Odor, and O3 Inside the Poultry Barn
Source: Front Chem. 2020 Jul 31;8:613. doi: 10.3389/fchem.2020.00613 (PMC7438853; doi:10.3389/fchem.2020.00613)
Supplement: Supplementary file 1 [file Data_Sheet_1.pdf]

Supplementary Material

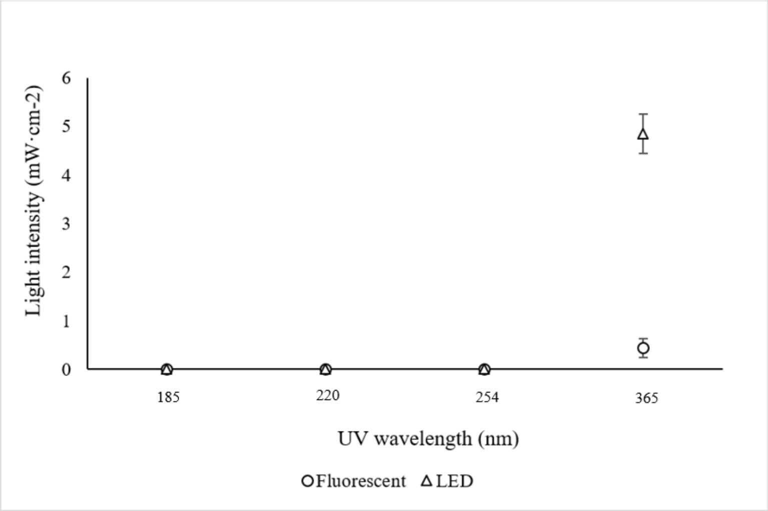

Supplementary Figure A1. The measured light intensity with different UV wavelength sensors. The distance from the lamp to the sensor: 20 cm.

Supplementary Table A2. NH_3 concentration (ppm) under different light types and treatment time. Values in the table report average concentration ± standard deviation.

Treatment time (s)	Items	Photolysis		Photocatalysis	
		Fluorescent	LED	Fluorescent	LED
40	Control	20.8 ± 0.4	20.3 ± 0.6	20.1 ± 0.1	21.6 ± 0.4
	Treatment	20.5 ± 0.9	20.3 ± 0.2	20.1 ± 0.5	21.0 ± 0.2
100	Control	21.3 ± 0.8	27.4 ± 0.3	20.4 ± 0.3	27.0 ± 0.8
	Treatment	21.1 ± 0.5	27.1 ± 0.4	19.9 ± 0.6	24.8 ± 1.4
170	Control	23.5 ± 0.6	20.9 ±0.6	23.9 ± 0.8	23.9 ± 1.5
	Treatment	23.3 ± 0.7	20.6 ± 0.4	22.6 ± 0.6	21.8 ± 1.2

Supplementary Table A2. N_2O concentration (ppm) under different light types and treatment time. Values in the table report average concentration ± standard deviation.

Treatment time (s)	Items	Photolysis		Photocatalysis	
		Fluorescent	LED	Fluorescent	LED
40	Control	0.22 ± 0.00	0.30 ± 0.02	0.28 ± 0.00	0.27 ± 0.01
	Treatment	0.22 ± 0.00	0.29 ± 0.00	0.28 ± 0.01	0.25 ± 0.00
100	Control	0.32 ± 0.01	0.30 ± 0.01	0.31 ± 0.00	0.28 ± 0.00
	Treatment	0.31 ± 0.01	0.29 ± 0.01	0.29 ± 0.00	0.26 ± 0.00

170	Control	0.33 ± 0.01	0.30 ± 0.00	0.31 ± 0.01	0.27 ± 0.01
	Treatment	0.32 ± 0.01	0.27 ± 0.01	0.29 ± 0.01	0.24 ± 0.01

Supplementary Table A3. Comparison of odor unit ($OU_e \cdot m^{-3}$) under different light types and treatment time. Values in the table report average concentration ± standard deviation.

Retention time(s)	Items	Photolysis		Photocatalysis	
		Fluorescent	LED	Fluorescent	LED
40	Control	555.1 ± 6.79	263.6 ± 14.1	541.4 ± 89.1	672.2 ± 47.4
	Treatment	554.8 ± 40.7	258.0 ± 19.3	504.8 ± 35.4	566.7 ± 79.2
100	Control	768.0 ± 85.6	722.8 ± 40.0	672.2 ± 15.2	740.1 ± 20.2
	Treatment	652.7 ± 41.2	837.7 ± 47.0	609.9 ± 44.6	686.2 ± 27.6
170	Control	616.7 ± 34.5	618.5 ± 50.1	626.7 ± 39.3	581.8 ± 24.7
	Treatment	576.8 ± 57.4	559.0 ± 106.5	579.2 ± 45.7	474.9 ± 37.8

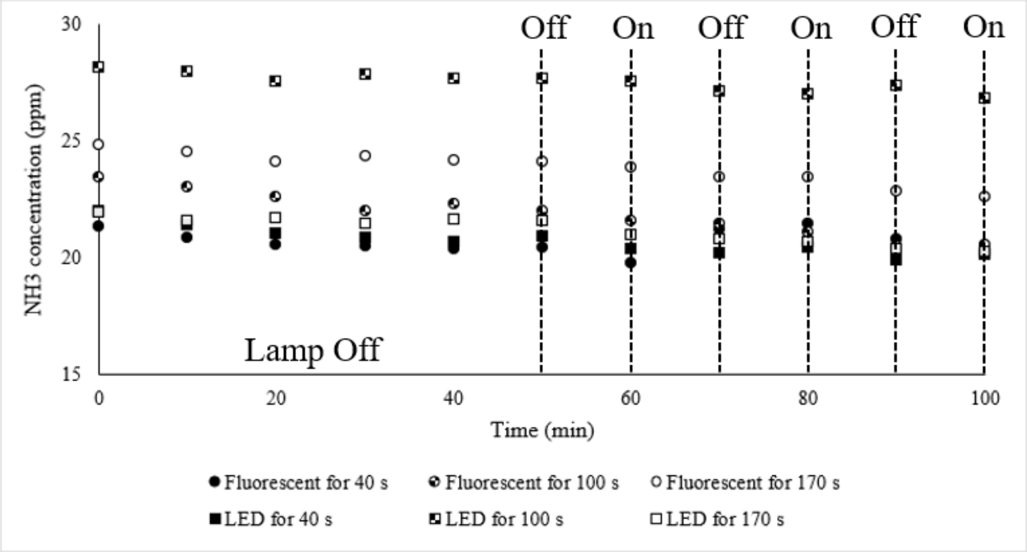

Supplementary Figure A2. Change of NH_3 concentration under the photolysis condition.

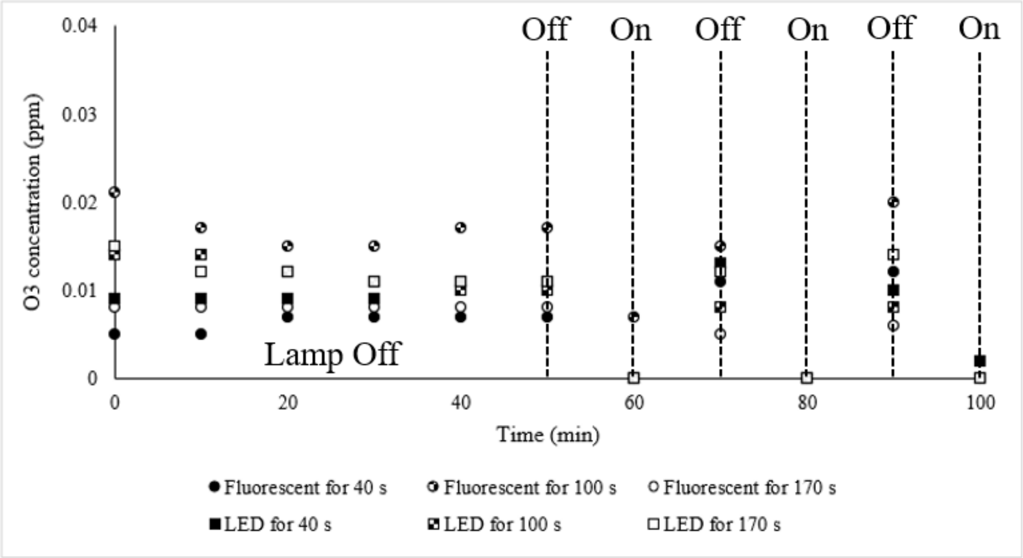

Supplementary Figure A3. Change of O_3 concentration under the photolysis condition.

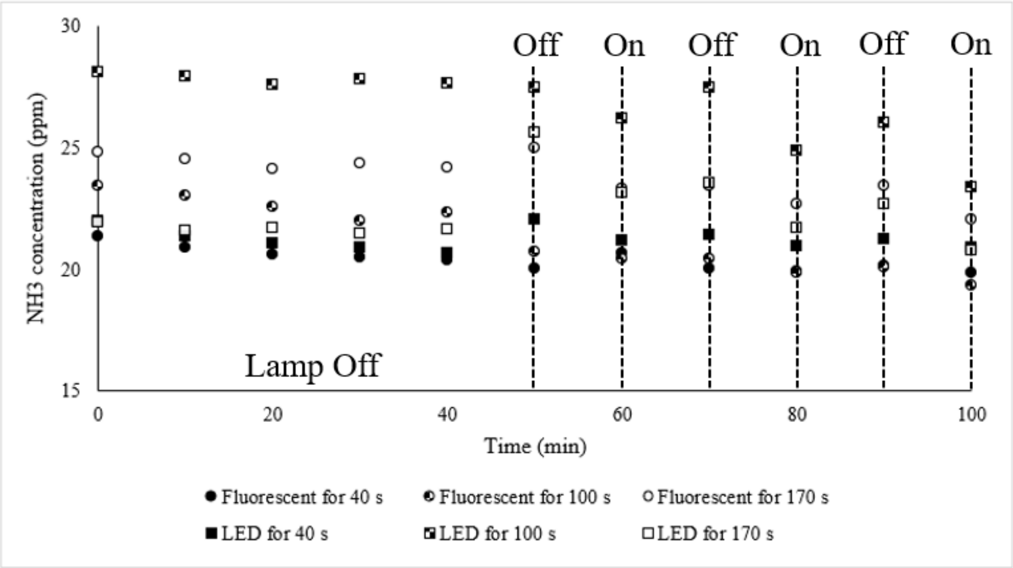

Supplementary Figure A4. Change of NH_3 concentration under the photocatalysis condition.

Supplementary Table A4. Changes of NH_3 concentration and % reduction under photocatalysis in three consecutive experiments illustrated in Figure 4A (representing statistically significant treatment).

	First trial			Second trial			Third trial		
UV-A type	Fluorescent	LED	LED	Fluorescent	LED	LED	Fluorescent	LED	LED
T time (s) [1]	170	100	170	170	100	170	170	100	170
Control (ppm)	25.0	27.5	25.6	23.5	27.5	23.5	23.4	26.0	22.7
Treatment (ppm)	23.3	26.2	23.1	22.7	24.9	21.7	22.1	23.3	20.8
% reduction	6.5	4.5	9.8	3.3	9.5	7.7	5.8	10.4	8.5

Note: [1] treatment time.

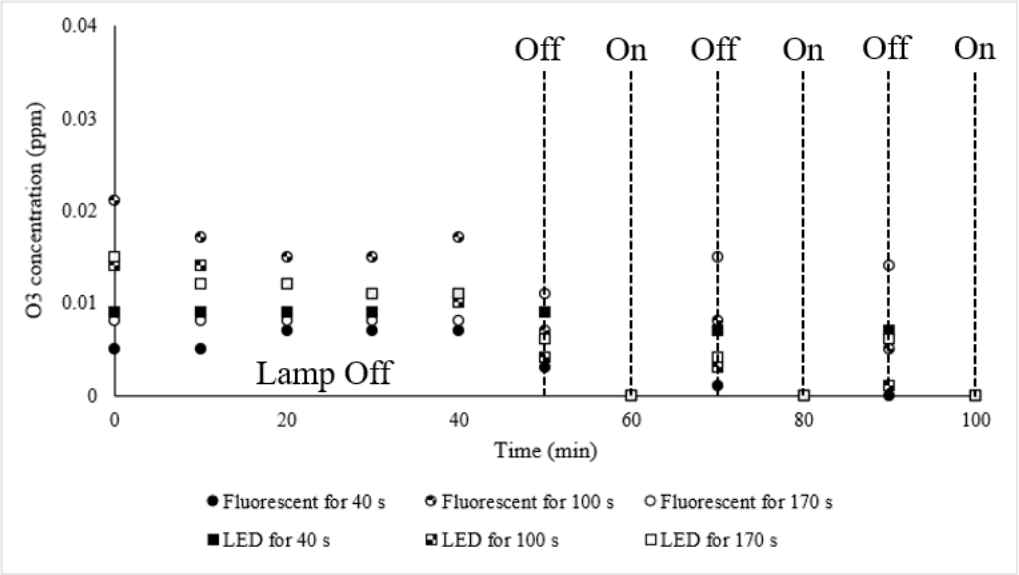

Supplementary Figure A5. Change of O_3 concentration under the photocatalysis condition.

Supplementary Table A5. Changes of O_3 concentration and % reduction under photocatalysis in three consecutive experiments illustrated in Figure 5A (A repeated reduction of 100% was achieved in all photocatalysis treatment conditions).

	First trial		Second trial		Third trial	
UV-A type	Fluorescent	LED	Fluorescent	LED	Fluorescent	LED
Treatment time (s)	170	170	170	170	170	170
Control (ppm)	0.011	0.006	0.015	0.004	0.014	0.006
Treatment (ppm)	0	0	0	0	0	0
% reduction	100	100	100	100	100	100
